# Evidence for a Lack of a Direct Transcriptional Suppression of the Iron Regulatory Peptide Hepcidin by Hypoxia-Inducible Factors

**DOI:** 10.1371/journal.pone.0007875

**Published:** 2009-11-18

**Authors:** Melanie Volke, Daniel P. Gale, Ulrike Maegdefrau, Gunnar Schley, Bernd Klanke, Anja-Katrin Bosserhoff, Patrick H. Maxwell, Kai-Uwe Eckardt, Christina Warnecke

**Affiliations:** 1 Department of Nephrology and Hypertension, University Hospital Erlangen, University of Erlangen-Nuremberg, Erlangen, Germany; 2 Department of Medicine, Rayne Institute, University College London, London, United Kingdom; 3 Institute of Pathology, University of Regensburg, Regensberg, Germany; L' Istituto di Biomedicina ed Immunologia Molecolare, Consiglio Nazionale delle Ricerche, Italy

## Abstract

**Background:**

Hepcidin is a major regulator of iron metabolism and plays a key role in anemia of chronic disease, reducing intestinal iron uptake and release from body iron stores. Hypoxia and chemical stabilizers of the hypoxia-inducible transcription factor (HIF) have been shown to suppress hepcidin expression. We therefore investigated the role of HIF in hepcidin regulation.

**Methodology/Principal Findings:**

Hepcidin mRNA was down-regulated in hepatoma cells by chemical HIF stabilizers and iron chelators, respectively. In contrast, the response to hypoxia was variable. The decrease in hepcidin mRNA was not reversed by HIF-1α or HIF-2α knock-down or by depletion of the HIF and iron regulatory protein (IRP) target transferrin receptor 1 (TfR1). However, the response of hepcidin to hypoxia and chemical HIF inducers paralleled the regulation of transferrin receptor 2 (TfR2), one of the genes critical to hepcidin expression. Hepcidin expression was also markedly and rapidly decreased by serum deprivation, independent of transferrin-bound iron, and by the phosphatidylinositol 3 (PI3) kinase inhibitor LY294002, indicating that growth factors are required for hepcidin expression *in vitro*. Hepcidin promoter constructs mirrored the response of mRNA levels to interleukin-6 and bone morphogenetic proteins, but not consistently to hypoxia or HIF stabilizers, and deletion of the putative HIF binding motifs did not alter the response to different hypoxic stimuli. In mice exposed to carbon monoxide, hypoxia or the chemical HIF inducer *N*-oxalylglycine, liver hepcidin 1 mRNA was elevated rather than decreased.

**Conclusions/Significance:**

Taken together, these data indicate that hepcidin is neither a direct target of HIF, nor indirectly regulated by HIF through induction of TfR1 expression. Hepcidin mRNA expression *in vitro* is highly sensitive to the presence of serum factors and PI3 kinase inhibition and parallels TfR2 expression.

## Introduction

The iron regulatory peptide hepcidin (gene name also: *HAMP*) is a liver-derived acute phase peptide and a key regulator of systemic iron metabolism [1], [2], [3], [4]. Hepcidin triggers internalization and degradation of the cellular iron exporter ferroportin in the intestinal epithelium and cells of the reticuloendothelial system thus reducing intestinal iron absorption and release of iron from body iron stores [5], [6]. High hepcidin serum levels are thus associated with reduced availability of iron for the hematopoietic system and an inadequate hematopoietic response even in the presence of appropriate erythropoietin (EPO) levels and iron supplementation therapy, features characteristic of the anemia of chronic disease (ACD). Therefore, novel therapeutic strategies leading to both increased serum EPO levels as well as reduced hepcidin expression may offer clinical benefit in the management of ACD.

Iron overload, infection and inflammatory cytokines are well recognized as factors leading to increased hepatic hepcidin expression [3], [7], [8]. Interleukin-6 (IL-6)-stimulated hepcidin induction is mediated by a highly conserved STAT3 binding element in the proximal promoter of the *HAMP* gene [9], [10]. This sequence motif controls both IL-6-induced, as well as basal, *HAMP* promoter activity. However, other signalling pathways also contribute to hepcidin regulation since IL-6 knock-out mice still induce hepcidin in response to endotoxin injection [11]. Much information about the determinants controlling hepcidin expression was obtained from the genetics of hereditary hemochromatosis [12], which is characterized by insufficient hepcidin levels due to mutations in the *transferrin receptor 2* (*TfR2*) gene, the hemochromatosis genes *HFE* and *HFE2* (also designated *hemojuvelin* = *HJV*), or the *hepcidin* gene itself. HFE2/HJV was shown to be a co-receptor for bone morphogenetic protein 2 (BMP-2), a protein of the TGF-β superfamily, which activates SMAD transcription factors that transactivate the *HAMP* promoter [13]. Liver-specific *SMAD4* (the common downstream mediator for all TGF-β superfamily ligands) knock-out mice exhibit marked iron accumulation and fail to increase hepcidin expression in response to TGF-β1, BMP-4, IL-6 or iron overload, suggesting a common role for SMAD4 in the manifold pathways of hepcidin regulation [14]. The conserved region of the *HAMP* promoter contains several putative binding sites for SMAD4 and BMP receptor-activated SMADs [13].

Other transcription factor binding sites suggested to contribute to basal as well as iron-overload induced hepcidin expression are a C/EBPα binding element and the upstream stimulatory factor (USF) binding site/E-box in the proximal promoter [15], [16].

Previous studies on hepcidin regulation have reported a decrease of hepcidin expression in response to hypoxia and anemia [17]. The heterodimeric hypoxia-inducible transcription factor (HIF) is the master regulator of the systemic and cellular adaptation to hypoxia. In the presence of molecular oxygen, the HIF-α subunit is hydroxylated by specific oxygen-, iron- and 2-oxoglutarate-dependent prolyl hydroxylases (PHDs), which is prerequisite for binding of the von Hippel-Lindau (VHL) protein, the recognition component of an ubiquitin ligase complex that targets HIF-α for proteasomal degradation. Under hypoxia, HIF-α is stabilized, translocates to the nucleus and binds as a dimer with the constitutive β-subunit and transcriptional co-activators to the hypoxia-responsive elements in the promoters or enhancers of its target genes (for review, see [18], [19], [20]). Amongst others, HIF transactivates enzymes of anaerobic glycolysis, glucose transporters, angiogenic factors and proteins involved in iron metabolism and erythropoiesis such as transferrin, transferrin receptor 1 and erythropoietin.

New pharmacological PHD inhibitors, which cause stabilisation of HIF and increased erythropoietin production, are currently undergoing clinical trials for the treatment of renal anemia. Intriguingly, these agents have been shown to suppress serum hepcidin levels in animal models (Langsetmo, I., *et al.* (2006) FG-2216 corrects anemia and improves iron utilization in a rat model of anemia of chronic disease: comparison to darbepoetin. Keystone Symposium ‘Hypoxia and Development, Physiology and Disease’. Breckenridge, CO, USA. pp. abstr. 247.; Seeley, T., *et al.* (2006) FG-2216: Tumor progression studies and correction of anemia of cancer in xenograft models. Keystone Symposium ‘Hypoxia and Development, Physiology and Disease’. Breckenridge, CO, USA. pp. abstr. 328). Furthermore, hepatocyte-specific HIF-1α knock-out in mice was associated with a markedly attenuated down-regulation of hepcidin expression under low iron diet in comparison with wildtype mice [21]. Stimulated by these studies we investigated the mechanisms underlying hypoxic hepcidin regulation and asked whether HIF is involved in hepcidin suppression. Our results show that hepcidin suppression is neither directly mediated by HIF nor indirectly through induction of transferrin receptor 1 (TfR1). Hepcidin expression *in vitro* strongly depends on serum factors and parallels TfR2 expression.

## Methods

### Cell Culture

Human hepatoma HepG2 cells were purchased from the German Collection of Microorganisms and Cell Cultures (DSMZ, Braunschweig, Germany). Human hepatoma Huh7 cells were a kind gift from Prof. Martina Muckenthaler, University of Heidelberg, Germany. Cell culture media and reagents were from PAA Laboratories (Coelbe, Germany). HepG2 and Huh7 cells were grown in Dulbecco's modified eagle medium with 1.0 g respectively 4.5 g glucose/l, 10% fetal calf serum (FCS), 2 mM L-glutamine, 100 U penicillin and 100 µg streptomycin per ml, and maintained at 37°C in a humidified 5% CO_2_ incubator.

### Stimulation Protocols

Cells were exposed to hypoxia (1% O_2_, 5% CO_2,_ 94% N_2_) in a HeraCell 150 hypoxic incubator (Thermo Electron) or stimulated with the iron chelator and HIF stabilizer 2,2′dipyridyl (DP, 100 µM; ICN Biomedicals, Irvine, CA, USA), the iron-independent hydroxylase inhibitor and HIF stabilizer dimethyloxalylglycine (DMOG, 1 mM; Frontiers Scientific Europe, Carnforth, Lancashire, U.K.) or the iron chelator desferrioxamine (DFO, 100 µM; Sigma-Aldrich, St. Louis, MO, USA) for 16 h–18 h if not indicated otherwise. IL-6 (2–10 ng/ml) and BMP-2 (100 ng/ml) were purchased from Hiss Diagnostics, Freiburg, Germany. To identify kinase pathways that were driving basal hepcidin expression in cultured cells in the presence of FCS, the following inhibitors were used: the phosphatidylinositol 3 kinase (PI3K) inhibitor LY294002 (10 µM, Calbiochem), the p38 stress-activated protein kinase (SAPK) inhibitor SB202190 (10 µM, Sigma), the mitogen-activated protein kinase kinase (MEK)1/2 inhibitor UO126 (1 µM, Cell Signaling) and the pan kinase inhibitor staurosporine (0.5 µM, Sigma).

### HIF Activation by Hypoxia, Carbon Monoxide or Chemical HIF Inducers in Mice

Mice livers were obtained from control animals of a study designed to investigate the protective effects of HIF activation on a subsequent kidney injury. These animal experiments were approved by the institutional review board for the care of animal subjects (Regierung von Mittelfranken, registration no.: 54-2531.31-25/06) and performed in accordance with National Institutes of Health guidelines and the German Animal Welfare Act. Six-weeks-old Balb/c mice (Charles River, Germany) were fed on a normal diet (‘Altromin 1324’, Altromin, Germany) and exposed to 8% O_2_/92% N_2_ for 8 h or to 8% O_2_/92% N_2_ for 8 h followed by 16 h at 10% O_2_/90% N_2_. Alternatively, mice were treated with 0.1% carbon monoxide in normal air for 6 to 8 h. Subsequently mice were killed by cervical dislocation and the livers were removed and immediately snap-frozen in liquid nitrogen. In an alternative approach, 6-weeks old male C57BL/6 mice were injected twice with the chemical HIF inducer *N*-oxalylglycine (OG, 9.2 mg/animal per injection; Frontiers Scientific Europe, Carnforth, Lancashire, U.K.) and sacrificed 16 hours after the second injection (24 h OG +16 h OG). In the second group, mice were injected with vehicle (tris-buffered saline) 24 hours after the OG treatment (24 h OG +16 h Tris). Control mice received vehicle only (24 h Tris +16 h Tris). Liver samples were immediately snap-frozen on liquid nitrogen and RNA was prepared for RNase protection assay or quantitative RT PCR as described below.

### RNA Preparation and RNase Protection Assay

Total RNA was prepared from cell cultures and mouse livers using RNABee™ (Biozol). Templates for RNA probes for human HAMP and mouse HAMP-1, human nucleoporin 98, TfR1 and TfR2 were generated by reverse transcriptase PCR (see **Table 1A** for primer sequences). Amplified fragments were cloned into the pcDNA3 vector (Invitrogen). Probes for U6 small nuclear RNA (U6sn), IGFBP1 and AngPTL4 were described before [22]. RNase protection assays were carried out as described previously [22], [23]. Quantification of signals was performed using a Phosphoimager (FujiBAS 2000, Fuji) and the AIDA™ image analysis software (Raytest).

**Table 1:** Primer sequences.

	A. Primers used for cloning of RNase protection assay probes
**human HAMP**	acc. no. NM_021175.2
hHAMP.Kpn+	5′AGCGGTACCAGTGGCTCTGTTTTCCCAC
hHAMP.Xho−	5′GTCCTCGAGCACATCCCACACTTTGATCG
**mouse HAMP-1**	acc. no. NM_032541.1
mHAMP.Kpn+	5′CTCGGTACCCAGGCTGCCTGTCTCCTG
mHAMP.Xho−	5′CACCTCGAGCAGAAGATGCAGATGGGGAAG
**human nucleoporin 98**	acc. no. U41815
NUP98.Kpn+	5′TTTGGTACCAGTTCATTTAGCCAGGC
NUP98.Xho−	5′TCCCTCGAGTCCTCCACTGCTAGTACTG
**human transferrin receptor 1**	acc. no NM_003234
hTfR1.Kpn+	5′GCAGGTACCGAGTCTCCAGTGAGG
hTfR1.Xho−	5′CTTCTCGAGATCCAGCCTCACGAGG
**human transferrin receptor 2**	acc. no. NM_003227
hTfR2.Kpn+	5′ACCGGTACCTGGTCCTGACGGCCCTG
hTfR2.Xho−	5′GGCCTCGAGGTCGCTCCAGTAGAGTCTG
	**B. Primers used for cloning of HAMP promoter constructs**
**construct HAMP.prom**	acc. no. NT 011109.15/Hs 1911266
HAMP.prom+	5′TTACTCGAGCCACATCTCAAGGGTCTGAC
HAMP.prom−	5′TGCAAGCTTGCCGTCTGTCTGGCTGTCC
**construct HAMP.prom.CpG**	acc. no. NT 011109.15/Hs 1911266
HAMP.CpG+	5′CCTGGTACCGCTGGGGGCTGCTCCTGTGT
HAMP.CpG−	5′TGGCTCGAGTAACTGGAAAATGTTTGAGCAAAG
**construct HAMP.promΔHRE**	acc. no. NT 011109.15/Hs 1911266
HAMP.promΔHRE	5′GTGTCTCGAGAGCTTAAAGCAATGGATGC
	**C. Primers used for quantitative RT PCR**
**human HAMP**	Hs_00221783_m1 (Applied Biosystems)
**18S RNA**	Hs_99999901_s1 (Applied Biosystems)
**human BMP-2**	acc. no. NM_001200
BMP-2+	5′GACACTGAGACGCTGTTCC
BMP-2−	5′CCATGGTCGACCTTTAGG
**human BMP-4**	acc. no. NM_130851.2
BMP-4+	5′GCCGGAGGGCCAAGCGTAGCCCTAAG
BMP-4−	5′CTGCCTGATCTCAGCGGCACCCACATC
**human BMP-6**	acc. no. NM_001718.4
BMP-6+	5′AAGGCTGGCTGGAATTTGACATCACG
BMP-6−	5′GGTAGAGCGATTACGACTCTGTTGTC
**human HAMP**	acc. no. NM_021175.2
hHAMP+	5′CCACAACAGACGGGACAACTT
hHAMP−	5′GGTTCTACGTCTTGCAGCACA
**mouse HAMP-1**	acc. no. NM_032541.1
mHAMP-1+	5′CCTATCTCCATCAACAGATG
mHAMP-1−	5′AACAGATACCACACTGGGAA
**mouse HIG2**	acc. no. AF141311
mHIG2+	5′ACCGTCGCCATGAAGTTCATGC
mHIG2−	5′CCTTAGGAGGCTGTGTGTTGG

### SiRNA Transfections

Cells were transfected with HIF-1α, HIF-2α or control siRNAs (luciferase (2 luc, 3 luc) or green fluorescent protein (GFP)) at a final concentration of 50 nM as described before [22], [23]. For TfR1 knock-down three independent siRNAs were used (sense strands): TfR1_A 5′GGAAUAAGGCCUUAAUAUG, TfR1_B 5′GGUACAACAGCCAACUGCU, and Hs_TFRC_5 no. SI00301896 (5′AAGUAGAUGGCGAUAACAGUC, Qiagen).

### Immunoblotting

Immunoblotting for HIF-1α (rabbit antiserum NB100–449, Novus Biologicals, USA), HIF-2α (mouse monoclonal antibody (mab) NB100–132) and transferrin receptor 1 (mab DF1513, Sigma) was performed as described previously [24]. Immunostaining for β-actin (mab AC74, Sigma) or α-tubulin (mab DM1A, Sigma) served as loading control.

### Reverse Transcriptase PCR (RT PCR)

Hepcidin mRNA expression in HepG2 and Huh7 cells was also quantified by RT PCR. Primers used were commercially available Taqman primers/probes from Applied Biosystems, or were designed using the NCBI primer design software. Hepcidin mRNA levels were related to 18S RNA. Expression analyses of BMP-2,-4 and -6 in Huh7 and HepG2 cells were performed by quantitative RT PCR in a LightCycler (Roche) as described before [25]. All primer sequences are listed in **Table 1C**.

### Cloning of Human *HAMP* Promoter Reporter Constructs

Three different hepcidin promoter fragments were amplified from genomic DNA by PCR using the primers given in **Table 1B** and Combizyme Polymerase Mix (Invitek), and cloned into the pGL2basic vector (Promega). The first construct (HAMP.prom) contained the proximal promoter, including the two putative HREs, from −nt 617 up to the translation start codon in the first exon. For a longer construct (HAMP.prom.CpG) a 1646 bp fragment containing the CpG island, which is located upstream of the core promoter, was fused 5′ to the 617-bp core promoter. In the third construct (HAMP.promΔHRE) a 145-bp region spanning the putative HREs was deleted from the core promoter. All plasmids were controlled by DNA sequencing.

### Luciferase Reporter Gene Assays

500 ng of the hepcidin promoter luciferase reporter plasmids were co-transfected with 50 ng of a pCMV-β-galactosidase expression vector in HepG2 or Huh7 cells by the use of Lipofectamine 2000 transfection reagents (Invitrogen). For HIF-α knock-down, 50 nM siRNAs were co-transfected with the plasmids as described before [24]. Cells were exposed to hypoxia, DP, DMOG, IL-6 or BMP-2 for 16 h, and subsequently lysed for determination of luciferase and β-galactosidase activities (Promega). Luciferase activities were normalized according to the respective β-galactosidase activities.

As control for the hypoxic induction a 6xHRE luciferase plasmid was used, which comprised six copies of the HRE of the *phosphoglycerate kinase* gene upstream of a *thymidin kinase* promoter. In some experiments a normoxically stable mouse HIF-1α triple mutant (mHIF-1αTM [24]) was co-transfected with the *HAMP* promoter constructs to determine the effect of HIF overexpression on *HAMP* promoter activity.

In HIF-α knock-down experiments 3 luc siRNA was used as negative control for the pGL2-based *HAMP* promoter constructs, and 2 luc siRNA as negative control for the pGL3 6xHRE construct [26].

### Analysis of TfR2 Protein Expression

TfR2 expression was determined by flow cytometry in HepG2 cells using the TfR2 mouse monoclonal antibody clone 353816 (R&D Systems).

### Statistical Analysis

Data are given as means±standard deviation (SD), if not indicated otherwise. Data were analysed using Student's t-test, Mann Whitney test and one-way ANOVA, as appropriate. A *p* value <0.05 was considered significant.

## Results

### Regulation of Hepcidin mRNA Expression by Hypoxia and Chemical HIF Inducers

**Figure 1:**
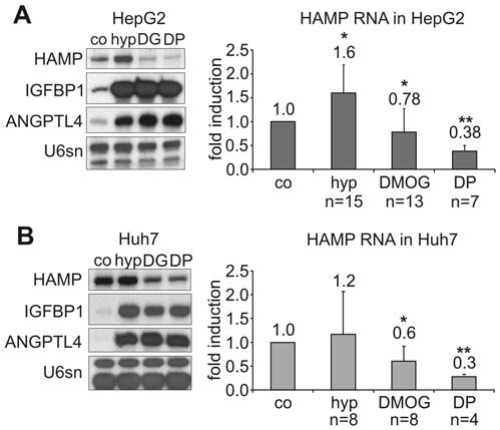
Response of hepcidin transcript levels to hypoxia and chemical HIF stabilization in human hepatoma cells. (**A**) RNase protection assays (RPA) demonstrating hepcidin (HAMP) mRNA regulation and HIF target gene (IGFBP1 and ANGPTL4) induction in HepG2 cells after 16 h exposure to hypoxia (1% O_2_), dimethyloxalylglycine (*abbreviated* DG *or* DMOG) or 2,2′dipyridyl (DP). (**B**) Hepcidin (HAMP), IGFBP1 and ANGPTL4 mRNA regulation in Huh7 cells. U6sn RNA served as loading control. Quantification was performed by phosphoimaging. Data are expressed as means±standard deviation (SD) of the indicated number of experiments; *p<0.05, **p<0.01 *vs.* unstimulated controls.

We first analyzed the response of hepcidin mRNA levels to hypoxia and chemical HIF stabilization in HepG2 cells. DP and DFO chelate Fe^2+^ and Fe^3+^, respectively, and inhibit iron- and 2-oxoglutarate (2-OG)-dependent dioxygenases, including the HIF PHDs and the asparagyl hydroxylase factor inhibiting HIF-1 (FIH-1), which leads to stabilization and activation of HIF even under normoxia. DMOG inhibits dioxygenases by competition with 2-OG [27]. While exposure to DP (100 µM) for 16 h robustly and consistently suppressed hepcidin mRNA levels, the response to hypoxia (1% O_2_) was variable and accompanied by an *increase* in hepcidin mRNA expression in 13 out of 15 experiments (**Figure 1A**). Exposure to DMOG (1 mM) was also associated with suppression of hepcidin mRNA, although in 3 out of 13 experiments hepcidin mRNA levels were slightly increased (**Figure 1A**). Transcription of known HIF target genes such as *IGFBP1* [28], [29] and *ANGPTL4* [30] were reliably induced by both hypoxia and chemical HIF stabilization (IGFBP1 in HepG2 cells: 19±8.5-fold by DMOG, 22.6±1.1-fold by DP, 15.3±5.5-fold by hypoxia; n = 7), verifying that HIF was activated under these conditions (**Figure 1A**).

Similar results were obtained in independently designed and performed experiments in a second laboratory: exposure to DFO (100 µM) reduced hepcidin mRNA (measured by reverse transcriptase real time quantitative PCR) to 7.6±7.1% (*p*<0.001, n = 8), whereas hypoxia again led to variable effects on hepcidin transcript expression with no difference in average transcript levels (103±80% of normoxic controls, n = 8). We first hypothesized that the characteristic cluster-like growth of HepG2 cells, which makes cell density difficult to control, may have confounded regulatory mechanisms. However, in human Huh7 hepatoma cells, which grow in monolayers, hepcidin mRNA expression was also highly variable under hypoxia, but significantly decreased by DMOG and DP, whereas established HIF target genes were consistently induced by hypoxia and chemical HIF activation (**Figure 1B**).

Since chemical HIF stabilizers also inhibit iron- and 2-OG-dependent dioxygenases other than the HIF PHDs and FIH-1, the observation that the hepcidin response differed between HIF activation by hypoxia and by chemical stabilization suggested that HIF was not the most important determinant of hepcidin expression in these experiments.

### Serum Deprivation Leads to a Marked Decrease of Hepcidin mRNA Levels

**Figure 2:**
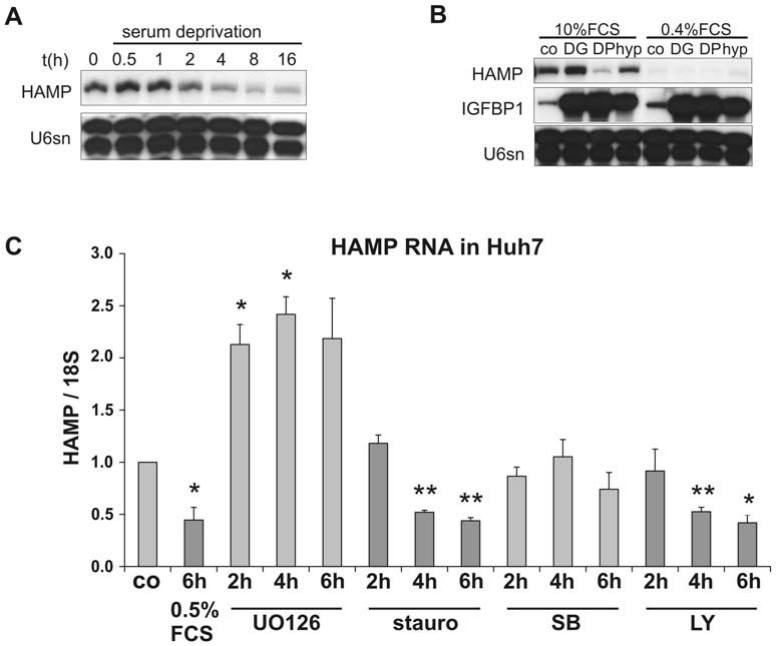
Response of hepcidin transcript levels to serum deprivation and protein kinase inhibition. (**A**) Serum withdrawal rapidly decreased hepcidin transcript levels in Huh7 cells. (**B**) After 40 h of FCS reduction from 10% to 0.4%, hepcidin transcripts were hardly detectable by RPA in HepG2 cells, whereas hypoxic IGFBP1 induction was not affected. Representative of three independent experiments. U6sn RNA served as loading control. (**C**) Exposure of Huh7 cells to protein kinase inhibitors revealed that the pan kinase inhibitor staurosporine (stauro; 0.5 µM) and the PI3 kinase inhibitor LY294002 (LY; 10 µM) reduced hepcidin expression similar to serum deprivation, whereas the p38 SAP kinase inhibitor SB202190 (SB; 10 µM) had no effect and the MEK1/2 inhibitor UO126 (1 µM) even increased HAMP/18S ratios. Data are results of qRT PCR analyses and given as means of three independent experiments±SEM; *p<0.05; **p<0.01.

Because consecutively performed experiments tended to show more similar hepcidin responses than those seen in widely separated experiments and the batches of the fetal calf serum (FCS) used for cell culture had been changed during this period, we investigated whether components of FCS could affect hepcidin expression. Serum deprivation from 10% to 0.4% rapidly reduced hepcidin mRNA levels in hepatoma cells. In Huh7 cells hepcidin transcript levels decreased to 54±8% after 2 h and 25±15% after 8 h of serum deprivation (*p*<0.05 vs. controls, n = 3; **Figure 2A**). After 40 h, hepcidin expression was almost completely eliminated as determined by RNase protection (**Figure 2B**). Quantification by phosphoimaging revealed that the effects of DP and DMOG on hepcidin mRNA expression were maintained in the absence of serum (0.65±0.04-fold by DMOG; 0.41±0.38-fold by DP), whereas the effect of hypoxia was not significant (1.23±0.3-fold; n = 4). Addition of transferrin-bound iron up to 2 mg/ml for 16 h neither prevented the decrease nor caused an increase in hepcidin levels (data not shown), which suggested that other components of the FCS were required to maintain basal hepcidin expression and that variability in the abundance of these factors in different batches of FCS may have contributed to the variability of hypoxic hepcidin expression between experiments. To further delineate the signalling pathways driving basal hepcidin expression in the presence of serum we used four different kinase inhibitors: the specific phosphatidylinositol 3 kinase (PI3K) inhibitor LY294002, which blocks growth factor-stimulated receptor tyrosine kinase signalling, the protein kinase inhibitor staurosporine, which inhibits protein kinase C and other kinases, the p38 α and β stress-activated protein kinase (SAPK) inhibitor SB202190, and the mitogen-activated protein kinase kinase (MEK)1/2 inhibitor UO126, which leads to a reduction of ERK1/2 activity and therefore also blocks signalling pathways of several growth factors. In Huh7 cells the kinase inhibitor staurosporine and the specific PI3 kinase inhibitor LY294002 significantly reduced hepcidin expression after 4 h and 6 h to about 50% and 40%, respectively, whereas UO126 intriguingly increased hepcidin expression after 2–6 h (**Figure 2C**). SB202190 had no effect in Huh7 cells. To test whether the increase by UO126 after 2 h was already caused by a counter-regulation, we also determined hepcidin mRNA levels after 30 and 60 min. Hepcidin mRNA increased 1.5±0.1-fold after 30 min and 1.8±0.2-fold after 60 min (n = 2, data not shown) suggesting that the observed effects were indeed direct effects. In HepG2 cells UO126 had comparable effects (not shown). These results indicate that PI3 kinase activity is required for basal hepcidin expression, whereas the MAP kinase pathway seems to suppress hepcidin expression in hepatoma cells.

### HIFs Are Not Necessary for the Modulation of Hepcidin mRNA Expression *In Vitro*

**Figure 3:**
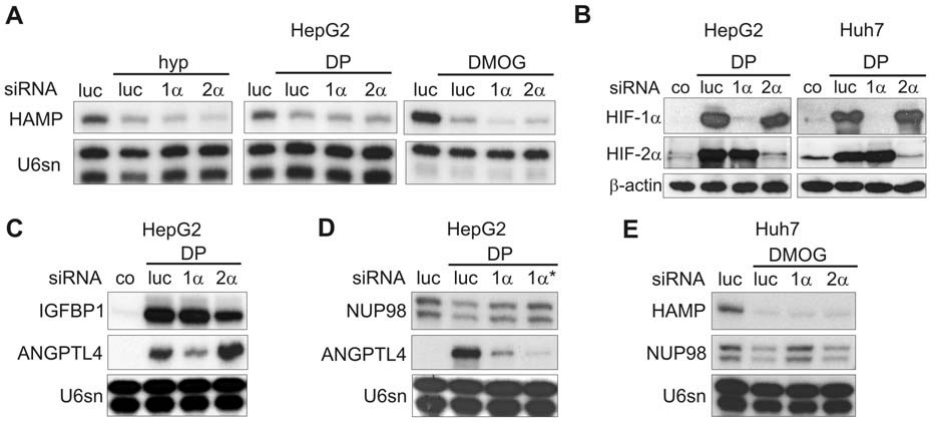
Suppression of hepcidin transcript levels is not mediated by HIF-1α or HIF-2α. (**A**) SiRNA-mediated knock-down of HIF-1α (1α) or HIF-2α (2α) did not attenuate the down-regulation of hepcidin mRNA levels in HepG2 cells after 16 h of stimulation; luciferase (luc) siRNA served as negative control. (**B**) Immunoblots demonstrating the efficiency of HIF-1α and HIF-2α knock-down in HepG2 and Huh7 cells. (**C**) mRNA expression of the established HIF target genes ANGPTL4 (HIF-1α target) and IGFBP1 (HIF-2α target in hepatoma cells) after HIF-α knock-down in HepG2 cells. (**D**) Two independent HIF-1α siRNAs (1α, 1α*) reversed the hypoxic down-regulation of the negatively regulated HIF-1 target NUP98. (**E**) In Huh7 cells hepcidin mRNA down-regulation by DMOG was not affected by HIF-α knock-down, whereas the decrease of NUP98 mRNA was reversed by HIF-1α knock-down. Results shown are representative of at least three independent experiments.

The transcriptional response to hypoxia is mediated predominantly by two HIF-α family members, HIF-1α and HIF-2α. To determine whether either of these HIF-α subunits is necessary for the modulation of hepcidin mRNA levels we transfected HepG2 cells with siRNAs directed against HIF-1α or HIF-2α. For each HIF-α subunit two different siRNAs were used. In no case was the knock-down of either HIF-α subunit accompanied by a rise in hepcidin mRNA (**Figure 3A**). Rather, hepcidin transcript levels appeared to be further reduced by HIF-1α knock-down (62±23% of luc siRNA-transfected, DMOG-stimulated HepG2 cells, *p*<0.05, n = 4), whereas HIF-2α siRNA had no significant effect (86±38% of luc siRNA-transfected cells). These results indicate that neither HIF-α subunit is required for the suppression of hepcidin by DMOG or DP. Even in those experiments where hypoxia was accompanied by reduced hepcidin mRNA levels, this suppression was also not abolished by HIF-α knock-down (**Figure 3A**). Moreover, combined knock-down of HIF-1α and HIF-2α did not have a significant effect on hepcidin expression (data not shown). Knock-down efficiencies were controlled by analyses of HIF-α protein expression (**Figure 3B**) and modulation of mRNA levels of IGFBP1 and AngPTL4 (**Figure 3C**), which were previously shown to be regulated in HepG2 cells by HIF-2α and HIF-1α, respectively [22]. In addition, down-regulation of nucleoporin 98 mRNA, a gene identified as a negative target of HIF-1α in a gene array study [22], was abrogated by HIF-1α knock-down in HepG2 and Huh7 cells, which demonstrated that HIF knock-down can also reverse HIF-mediated down-regulation of gene expression (**Figure 3D–E**).

### The Regulation of Hepcidin mRNA Expression by Hypoxia and Chemical HIF Activation May Not Be Solely Mediated by the Promoter Region

**Figure 4:**
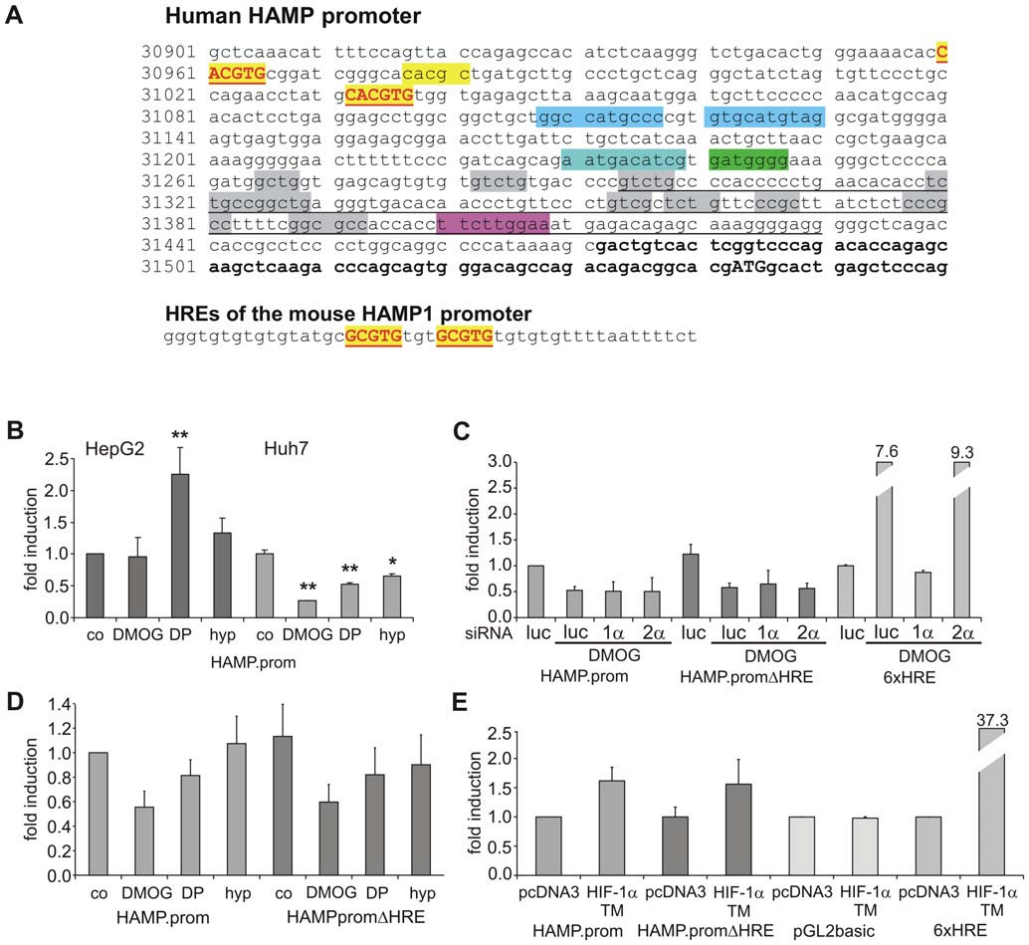
The hypoxic response of the *hepcidin* promoter is cell type-specific and independent of HIF. (**A**) Sequence of the human *hepcidin* promoter (acc. no. AD000684.1) with two putative HIF binding motifs which also conform to E-box/USF binding sites (yellow boxes, red letters) and one additional HIF binding site (yellow); binding sites for p53 (light blue), AP1 (blue-green), C/EBPα (green), STAT3 (pink), SMAD (grey) are also marked; mRNA sequence (bold), translation start codon (ATG); underlined sequence represents region highly conserved between human and murine *hepcidin 1* gene. Lower sequence, two putative HREs were identified in the mouse *hepcidin 1* gene about 2.1 kbp upstream to the transcription start. These HIF binding sites do not conform to E-box/USF binding site consensus sequence. (**B**) A 617-bp human *hepcidin* promoter construct (HAMP.prom) responded differently to hypoxia (hyp), DMOG and DP in HepG2 and Huh7 cells after 16 h of stimulation (co = control). Data are means±SD of five (HepG2 cells) or three (Huh7 cells) independent experiments. (**C**) SiRNA knock-down of HIF-1α (1α) or HIF-2α (2α) did not reverse the down-regulation of promoter activity by DMOG in Huh7 cells; a 6xHRE luciferase reporter served as control. 3 luc siRNAs served as negative control for the pGL2-based *hepcidin* promoter constructs and 2 luc siRNA as negative control for the pGL3 6xHRE; deletion of the putative HREs in the *HAMP* promoter (HAMP.promΔHRE) did not alter the response of the luciferase construct to DMOG nor did HIF-α knock-down. (**D**) Deletion of putative HREs (HAMP.promΔHRE) did not alter the response of the *hepcidin* promoter to DMOG, DP or hypoxia in Huh7 cells. (**E**) Overexpression of a stable HIF-1α triple mutant (HIF-1αTM) tended to increase *hepcidin* promoter activity in comparison with the empty vector control (pcDNA3). The 6xHRE reporter served as positive and the promoter-less pGL2basic vector as negative control, respectively; *HAMP* promoter activities given in B–D are means of three independent experiments±SD. *p<0.05; **p<0.01 *vs.* unstimulated control (co).

To investigate whether the effects of hypoxia and chemical HIF stabilization on hepcidin mRNA levels are transcriptional, we analyzed the human *hepcidin* promoter *in silico* and in reporter assays. Using Genomatix MatInspector software [31] we identified two putative hypoxia-responsive elements (HREs, CACGTG) 511 and 583 bp upstream to the start codon in exon 1, which also matched the E-box/USF binding site consensus sequence (CANNTG), and a further putative HRE on the antisense strand between the two HREs/E-boxes (**Figure 4A**). We hypothesized that HIF could act as a competitor at the congruent *cis*-active elements and displace positive regulators such as USF and c-myc under hypoxic conditions although in the murine promoter the HREs are not identical with these transcription factor binding sites.

To test our hypothesis, we cloned three fragments of the human *hepcidin* promoter in the pGL2basic luciferase vector: the first comprised a 617-bp core promoter fragment (HAMP.prom). The second contained, upstream to the core promoter, a 1635-bp fragment of the adjacent 5′flanking region which comprised a CpG island (HAMP.prom.CpG). The third construct was a deletion mutant of the core promoter which lacked the region encompassing the putative HREs (HAMP.promΔHRE).

Luciferase reporter assays in HepG2 and Huh7 cells revealed that the *hepcidin* promoter responded differently in the two cell types and furthermore, that this regulation did not correspond to the regulation at the mRNA level for all of the stimuli. Exposure to hypoxia, DMOG or DP of Huh7 cells transfected with the HAMP.prom construct resulted in the anticipated reduction of luciferase activity (**Figure 4B**). In contrast, HepG2 cells transfected with the HAMP.prom construct showed an increase in luciferase activity when exposed to DP and no significant change when exposed to hypoxia or DMOG. Despite these differences between cell types, the effects of HIF-α knock-down or removal of the putative HREs in the promoter constructs were identical: Neither knock-down of the HIF-α subunits nor deletion of the putative HREs altered the response to DMOG, DP or hypoxia (**Figure 4C–D**). Overexpression of a normoxically stable HIF-1α mutant was associated with mildly increased luciferase activity. However, this effect was also independent of the presence of the putative HREs in the HAMP promoter (**Figure 4E**). A 6xHRE luciferase construct, which is activated by endogenous HIF-1α, but not HIF-2α, was used as control for these experiments.

**Figure 5:**
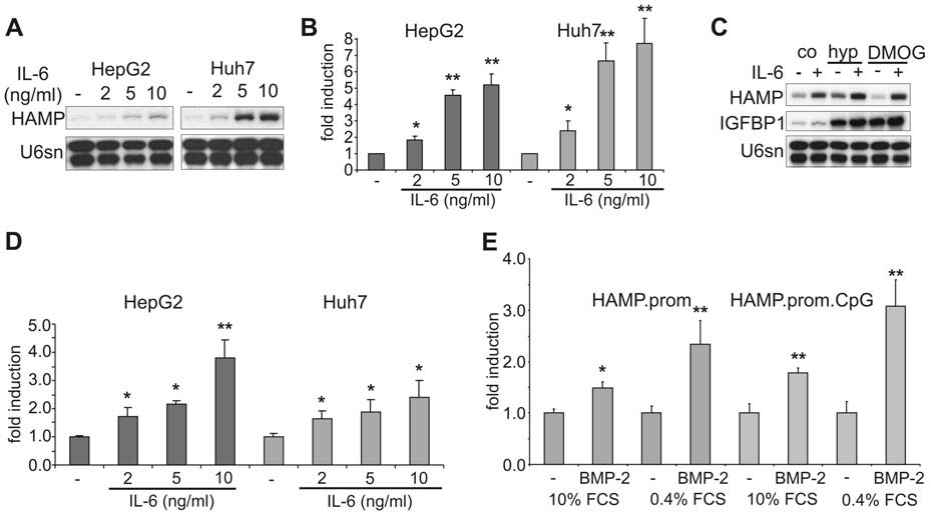
Transcriptional induction of hepcidin expression by IL-6 and BMP-2. (**A**) RNase protection assays demonstrating dose-dependent induction of hepcidin mRNA by IL-6 in HepG2 and Huh7 cells. U6sn RNA served as loading control. (**B**) Quantification of hepcidin mRNA induction; means±SD of three independent experiments; *p<0.05, **p<0.01 *vs.* unstimulated cells. (**C**) IL-6 blunted, but did not abrogate the down-regulation of hepcidin mRNA levels by DMOG. IGFBP1 mRNA served as control for the hypoxic stimulation. (**D**) IL-6 activated the human *hepcidin* promoter (HAMP.prom) in HepG2 and Huh7 cells. (**E**) Activation of the *hepcidin* promoter by BMP-2 (100 ng/ml) was mediated primarily by the proximal promoter, since fusion of the 5′adjacent CpG island to the proximal promoter (HAMP.prom.CpG) did not significantly alter the response to BMP-2. The activation was more pronounced under serum-reduced conditions (0.4% FCS). D–E, data are means±SD of three independent experiments; *p<0.05, **p<0.01 *vs.* unstimulated controls.

To confirm that the hepatoma cells responded to other known stimuli as previously reported and that the cloned hepcidin promoter fragments were functional, we stimulated HepG2 and Huh7 cells with IL-6 and BMP-2. Exposure to IL-6 for 16 h led to a significant dose-dependent increase of hepcidin mRNA levels in both cell lines (**Figure 5A–B**). We then determined the effects of a combined exposure to IL-6 and hypoxia or chemical HIF inducers. The induction by IL-6 may have been attenuated in HepG2 cells in the presence of DMOG, although this difference did not reach statistical significance (IL-6 2.7±0.9-fold, IL-6 plus DMOG 1.23±0.2-fold *vs.* unstimulated controls; n = 3, *p* = 0.056; **Figure 5C**). The moderate induction by hypoxia in these experiments was further enhanced by IL-6. In Huh7 cells, DMOG exposure completely abolished the induction of hepcidin by IL-6 (IL-6 3.8±1.5-fold, IL-6 plus DMOG 0.6±0.3-fold, n = 4; *p*<0.05; not shown). Compatible with the hypothesis that the promoter region mediates the hepcidin response to IL-6, the HAMP.prom luciferase reporter responded in a dose-dependent fashion to IL-6, similar to the endogenous mRNA (**Figure 5D**). Exposure to BMP-2, a further well characterized stimulus of hepcidin gene expression [13], [32], increased hepcidin promoter activity, although high concentrations (50–100 ng/ml) were required (**Figure 5E**). This effect was more pronounced in serum-starved cells (0.4% FCS) than on a 10% FCS background, which may be due to the contribution of BMPs in the FCS to baseline hepcidin expression, in keeping with the observation that serum deprivation leads to a rapid and marked decrease of hepcidin mRNA levels as shown in Figure 2. Thus, the induction of hepcidin by IL-6 and BMP-2 in the two hepatoma cell lines was reproducible, dose-dependent and mediated by the proximal promoter region, in agreement with previous studies [9], [10], [13], [33], [34], [35]. IL-6 stimulation was attenuated or abolished by DMOG exposure and this depended on both the cell type and the dose and timing of the two stimuli, suggesting that they employ independent signal transduction pathways.

### Transferrin Receptor 1 (TfR1/TFRC) Knock-Down Does Not Reverse Hepcidin Down-Regulation in Hepatoma Cells

**Figure 6:**
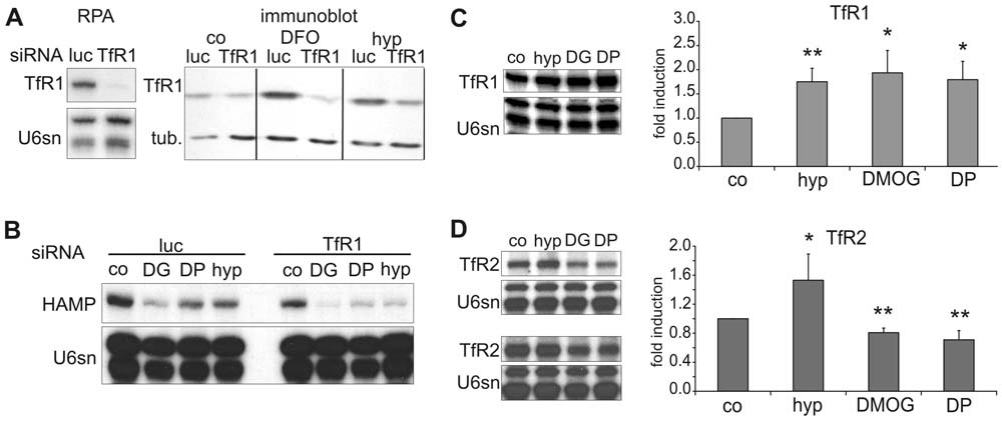
TfR1 is not necessary for hepcidin down-regulation, but TfR2 and hepcidin expression correlate. (**A**) TfR1 mRNA and protein levels were reduced by TfR1 siRNA transfection as determined by RNase protection assay (RPA) and immmunoblot; luciferase (luc) siRNA served as negative control; α-tubulin (tub) was used as loading control for the immunoblot. DFO = desferrioxamine (100 µM). (**B**) In Huh7 cells hepcidin mRNA down-regulation after 16 h of exposure to DMOG (DG), DP and hypoxia was not abrogated but rather enhanced by TfR1 knock-down; co = unstimulated control cells. Data are representative of at least two independent experiments in either cell line. (**C**) TfR1 mRNA induction by hypoxia, DMOG and DP in Huh7 cells, 40 µg total RNA were used per sample, n = 3–4. (**D**) TfR2 mRNA regulation in Huh7 cells (upper panel) and HepG2 cells (lower panel), 60 µg total RNA per sample; bar graph, quantification of TfR2 levels in Huh7 cells; n = 4–6; *p<0.05, **p<0.01.

The ubiquitously expressed TfR1 is believed to import transferrin-bound iron (Fe2Tf) and interacts with HFE in the absence of Fe2Tf, whereas TfR2 is suggested to act as a signal of extracellular Fe2Tf availability resulting in hepcidin expression, probably by activating the mitogen-activated protein kinases ERK1/ERK2 and p38 [36]. While TfR1 is moderately inducible by hypoxia and iron deficiency via HIF and iron regulatory proteins (IRPs), TfR2 expression is not known to be regulated by these stimuli. It thus appeared possible that upregulation of TfR1 expression by HIF or IRPs may lead to competition with TfR2 for Fe2Tf and HFE and thus contribute to suppression of hepcidin expression by reducing TfR2 signalling [37], [38]. We tested this hypothesis by siRNA-mediated knock-down of TfR1. Knock-down was performed using three independent siRNAs in two different laboratories and its efficiency was measured by RNase protection assay, immunoblot and FACS analyses. Knock-down amounted to about 90% at the mRNA and 70–85% (under induced conditions) at the protein level (**Figure 6A**). The decrease of hepcidin levels in the presence of hypoxia, iron chelation (Fe^2+^ by DP and Fe^3+^ by DFO) or chemical HIF stabilization by DMOG exposure was not blunted by TfR1 depletion, but rather enhanced (**Figure 6B** and data not shown). Thus, at least *in vitro*, induction of TfR1 expression does not appear necessary for the down-regulation of hepcidin transcription.

**Figure 7:**
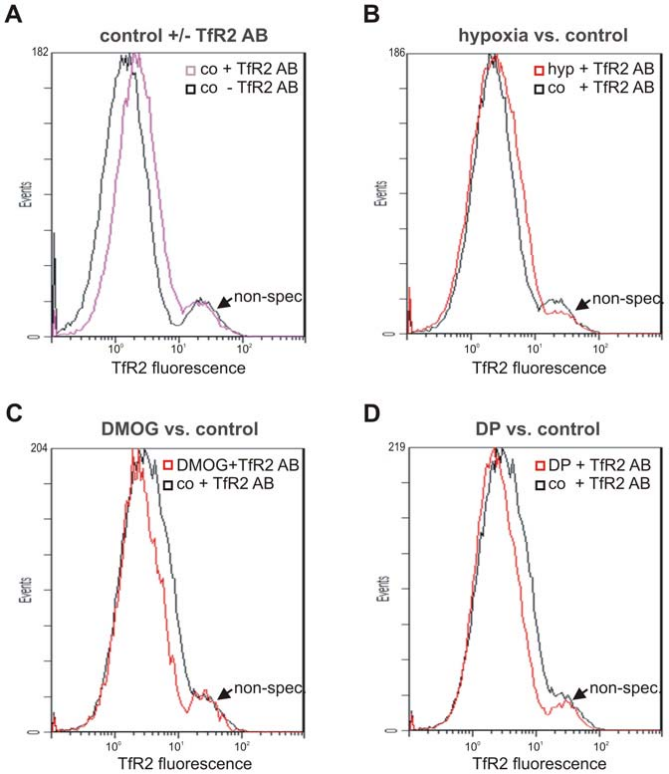
Effects of hypoxia, DMOG and DP on TfR2 protein expression in HepG2 cells. (**A**) FACS analysis of HepG2 cells with a TfR2 mouse monoclonal antibody demonstrating TfR2 expression on HepG2 cells under baseline conditions in comparison with an isotype-matched negative control antibody (co - TfR2). (**B**) After 16 h exposure to hypoxia a moderate induction was detected. After 16 h exposure to DMOG (**C**) or DP (**D**) TfR2 protein expression was reduced. C–D, representative of three independent experiments; B, representative of two out of three experiments.

We then compared mRNA expression of TfR1 and TfR2 after 16 h exposure to hypoxia, DMOG and DP. As anticipated based on its known regulation by HIF-1 and IRPs, TfR1 mRNA was moderately increased by hypoxia, DMOG and DP after 16 h (**Figure 6C**). Intriguingly TfR2 mRNA, which was less abundant than TfR1 mRNA in both hepatoma cell lines, was moderately increased by hypoxia, and reduced by DMOG and DP in Huh7 cells (**Figure 6D**, upper panel and bar graph). A similar pattern was observed in HepG2 cells, although the increase by hypoxia was somewhat less consistent than that seen in Huh7 cells (**Figure 6D**, lower panel). Flow cytometry with a monoclonal TfR2 antibody confirmed that in HepG2 cells the mRNA regulation by DP and DMOG was accompanied by a reduction of receptor protein on the cell surface to 66±11% and 79±4% (n = 3; p<0.05), respectively (**Figure 7**), whereas the increase by hypoxia was not significant. TfR2 expression on Huh7 cells was too low for flow cytometry.

Since this pattern of TfR2 regulation matched the modulation of hepcidin transcripts, in particular the reduction by DMOG and DP, regulation of TfR2 expression may contribute to the observed responses of hepcidin expression *in vitro*.

### Regulation of Endogenous BMP Expression Does Not Underlie the Suppression of Hepcidin by DMOG and DP

**Figure 8:**
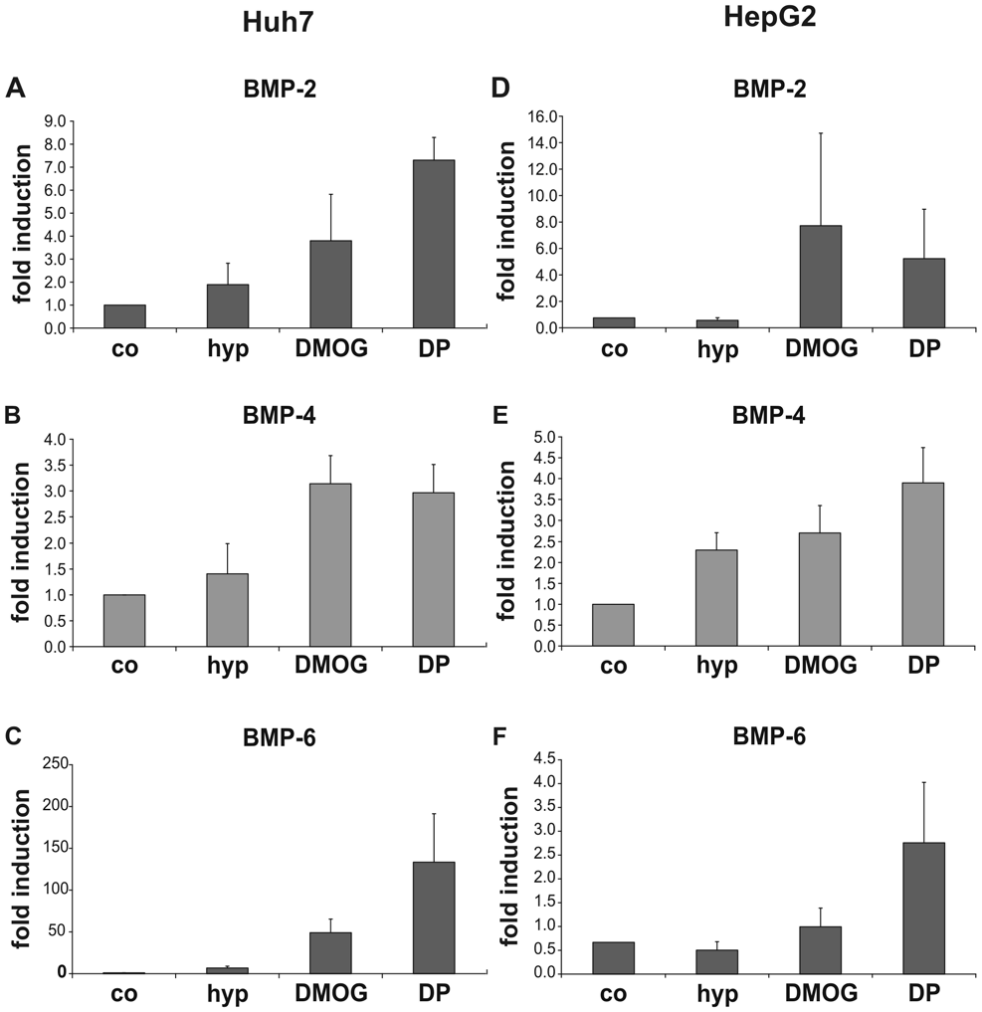
Regulation of BMP-2, -4 and -6 mRNA expression in hepatoma cells. Quantitative RT PCR analysis of BMP-2 (**A, D**), BMP-4 (**B, E**) and BMP-6 (**C, F**) mRNA expression in Huh7 (**A–C**) and HepG2 cells (**D–F**). Cells were exposed to 1% O_2_ (hyp), 100 µM DMOG or 100 µM DP for 16 hours. BMP mRNA levels were related to β-actin mRNA expression. Data given are means of two independent experiments and two RT PCRs per experiment.

Since hepcidin down-regulation by inhibitors of 2-OG-dependent dioxygenases was neither mediated by HIF nor by modulation of TfR1 expression, we further speculated that the expression of BMPs may have been altered by the experimental manipulations and may mediate hepcidin regulation. BMP-2, -4 and -6 have been shown to contribute to hepcidin expression in HepG2 cells [32] and BMP6 was recently shown to be essential for hepcidin expression *in vivo* [39], [40]. We therefore determined mRNA levels of these BMPs in hepatoma cells by reverse transcriptase quantitative PCR. However, in contrast to TfR2 mRNA, BMP transcript levels did not correlate with hepcidin expression. With the exception of BMP-2 and -6 in HepG2 cells, all three BMPs were slightly up-regulated by hypoxia and markedly up-regulated by DMOG and DP in both cell lines (**Figure 8**). Thus, modulation of hepcidin expression by endogenous BMPs does not seem to underlie the reduction of hepcidin mRNA by DMOG and DP.

### Short-Term Hypoxia Does Not Consistently Down-Regulate Liver Hepcidin 1 mRNA Expression in Mice

**Figure 9:**
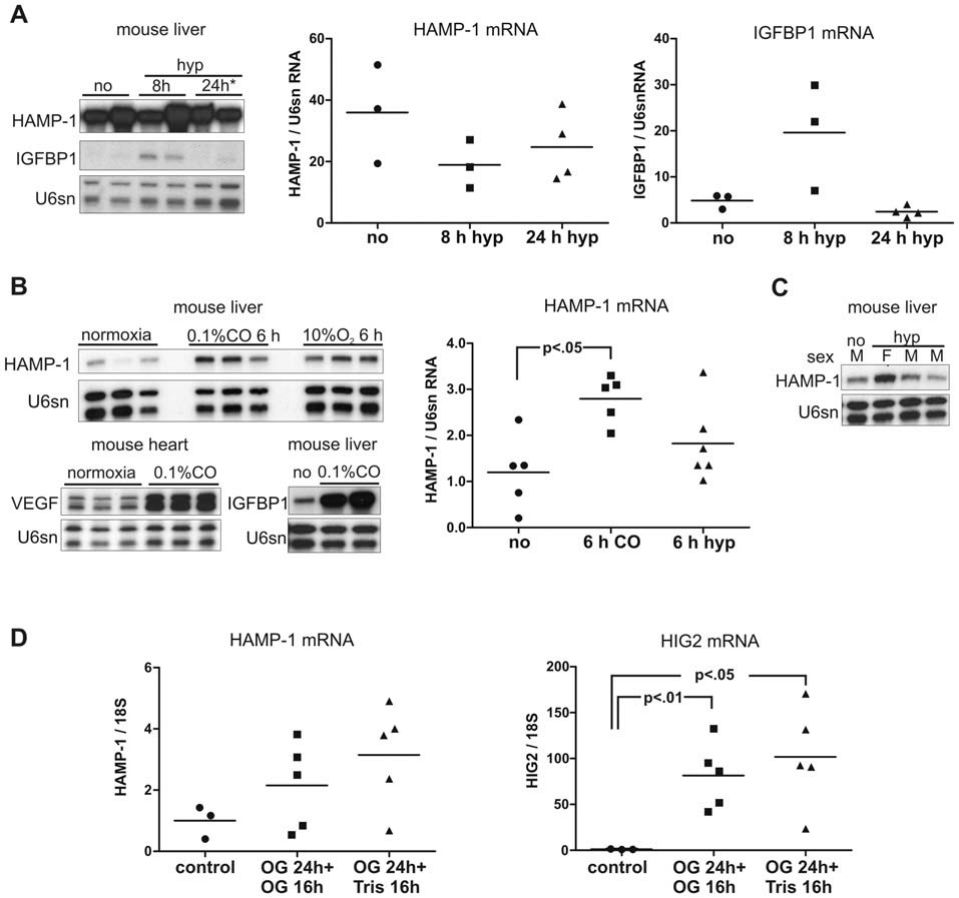
Hepcidin 1 transcript levels are not consistently down-regulated by hypoxia or carbon monoxide in mice. (**A**) Mice exposed to 8% O_2_ (hypoxia) for 8 h or 8 h 8% O_2_ followed by 16 h 10% O_2_ ( = 24 h) did not show significantly decreased liver hepcidin 1 (HAMP-1) mRNA expression; no = normoxia. Representative RPA results (left panel) and quantification of HAMP-1 and IGFBP1 transcript levels in the total numbers of animals used (right graphs). (**B**) 0.1% carbon monoxide (CO) for 6 h moderately increased hepcidin 1 transcript levels and markedly increased vascular endothelial growth factor (VEGF) and IGFBP1 expression. The effect of hypoxia was again not significant. Representative RPA results and quantification of HAMP-1 transcript levels in the total number of animals used. (**C**) Liver hepcidin mRNA levels of female (F) mice were higher than those of males (M). (**D**) In an alternative approach, male C57BL/6 mice were injected with the HIF inducer *N*-oxylylglycine (OG) twice (24 h OG +16 h OG; n = 5) or once, followed by a vehicle injection (24 h OG +16 h Tris; n = 5). The male control mice received vehicle only (24 h Tris +16 h Tris; n = 3). The left graph shows that in both treatment groups HAMP-1 mRNA levels were slightly, but not significantly increased 40 h after the first OG injection. The right graph shows that HIF was activated by OG in the livers, because mRNA of the HIF target gene HIG2 was significantly induced (indicated p values are Student's t-test; p<0.05 for either OG group vs. control in Mann Whitney test).

To find out whether the reported down-regulation of liver hepcidin mRNA levels in mice could be reproduced and inversely related to the induction of HIF target genes, Balb/c mice on a normal (iron-rich) diet were exposed for 6–8 h to hypoxia (8% O_2_, **Figure 9A–B**) or 0.1% carbon monoxide (CO, **Figure 9B**), which leads to functional anemia and HIF activation. Surprisingly, these conditions did not result in a consistent reduction of hepatic hepcidin 1 mRNA expression (8 h hypoxia vs. normoxia p = 0.2, 24 h hypoxia vs. normoxia p = 0.4 in Mann Whitney test). Hepcidin 1 mRNA levels were highly variable both under basal and stimulated conditions (mean HAMP-1/U6sn ratios±SEM normoxia 36±9.3, 8 h hypoxia 18.9±4.5, 24 h hypoxia 24.7±5.7). Since female mice show higher baseline hepcidin expression than males ([41]; **Figure 9C**), we used exclusively female littermates for the experiments. Even under these conditions, the response to hypoxia was variable and in the CO-treated animals we observed up- rather than down-regulation of hepatic hepcidin 1 mRNA. Mean HAMP-1/U6sn ratios±SEM were 0.28±0.022 in the CO-treated group vs. 0.12±0.036 in the control group (p<0.01 in Student's t-test, p<0.05 in Mann Whitney test; **Figure 9B**). The difference between the hypoxia-treated group (0.18±0.035) and the normoxic control group was not significant (Student's t-test and Mann Whitney test). To verify that the experimental interventions were sufficient to up-regulate HIF, expression of hepatic IGFBP1 and myocardial vascular endothelial growth factor-A (VEGF) were measured. All animals exposed to CO showed a marked induction of HIF target genes, whereas the response to hypoxia was less pronounced and no more detectable after 24 h hypoxia. One animal of the 8 h hypoxia group quantitated in the bar graph in Figure 9A did not show liver IGFBP1 induction, but also exhibited the lowest hepcidin 1 transcript level.

In a further experimental approach we used the chemical HIF inducer *N*-oxalylglycine (OG), the water-soluble derivative of DMOG, because chemical HIF induction is better tolerated during prolonged exposure than hypoxia or CO. *In vivo* OG is taken up by epithelial cells of the liver and kidney mediated by different organic anion transporters. Because EPO is produced by interstitial fibroblasts of the kidney, OG does not lead to EPO induction which was confirmed by RT PCR in pilot experiments (mean EPO/18S ratios±SEM of OG-treated mice: 0.32±0.34, of hypoxic mice: 35.4±22.2, of CO-exposed mice: 131.1±61.2, normoxic mice = 1; n = 3 for each group). Five male C57BL/6 mice were injected with 9.2 mg OG twice (24 h OG +16 h OG), another five animals received at first OG and after 24 h an injection with vehicle only (24 h OG +16 h Tris), whereas the three male control mice were injected with vehicle only. RNA was prepared from liver samples, and mRNA expression of HAMP-1 and HIG2, a HIF target gene [22], was determined by the use of quantitative RT PCR (**Figure 9D**). All OG-treated animals showed a pronounced increase of HIG2 mRNA expression, indicative of HIF activation in the liver (mean HIG2/18S ratios±SEM were 81.5±16.2 in the 24 h OG+16 h OG group compared with the control 1.0±0.18, p<0.01; mean HIG2/18S ratios were 101.7±24.4 in the 24 h OG+16 h Tris group, p<0.05 vs. controls as analyzed by Student's t-test, p<0.05 for either treatment group vs. controls in Mann Whitney test). Comparable to the results obtained with hypoxia and CO, HAMP-1 was slightly increased (n.s.), but not decreased, by OG treatment. Mean HAMP-1/18S ratios±SEM were 2.15±0.63 for the 24 h OG +16 h OG group, 3.15±0.74 for the 24 h OG +16 h Tris group and 1±0.31 for the control group (n.s. in Student's t-test, Mann Whitney test and one-way ANOVA).

Although performed on a limited number of animals, these data suggest that hepcidin regulation *in vivo* is complex and that, in the short term, HIF activation in the liver is not sufficient to suppress hepcidin.

## Discussion

### Hypoxia Does Not Reproducibly Lead to Hepcidin Down-Regulation *In Vitro* and *In Vivo*

The aim of the present study was to determine the contribution of HIF to the transcriptional regulation of hepcidin expression. In contrast to previous studies [17], [42], [43], our results show that hypoxia does not consistently suppress hepcidin expression in human hepatoma cells or, after short-term hypoxia, carbon monoxide exposure or *N*-oxalylglycine (OG) administration in mice on a normal iron-rich diet. Moreover, experiments with primary mouse hepatocytes also did not show a significant effect of hypoxia (1.19±0.4-fold induction *vs.* unstimulated controls, five independent cell preparations, data not shown). In contrast, the reduction of hepcidin mRNA levels by chemical HIF stabilizers, in particular the iron chelator DP, was reproducible, but not dependent on HIF-1α, HIF-2α, TfR1 induction or modulation of BMP expression. In those experiments which showed a *down*-regulation of hepcidin mRNA by hypoxia this was also not dependent on HIF. The variability of the hypoxic hepcidin response points at an overlay of several, presumably indirect effects of hypoxia on hepcidin expression.

Two previous studies confirmed the initially reported hepcidin down-regulation by hypoxia [42], [43], which may be due to different experimental conditions, such as the duration of hypoxic exposure (24 h *vs.* 16 h in our study) and the serum used for cell culture. Prolonged hypoxia triggers a broad, predominantly HIF-independent suppression of gene transcription, which may also account for the down-regulation of hepcidin. In accordance with our results, both studies demonstrated that the hypoxic down-regulation was independent of HIF.

The results of the animal studies in the present report were heterogeneous and did not show a consistent up- or down-regulation of hepcidin by short-term hypoxia (6–24 h) in Balb/c mice. Obviously, food intake and presumably also circadian rhythms, as it was observed in humans [44], prevail over the effects of hypoxia in mice. However, in two of three experiments we found rather up- than downregulation of hepcidin expression by hypoxia, carbon monoxide or the chemical HIF inducer OG (although the increase only reached statistical significance in the CO-treated group). In this respect, the results of a recent study that showed HIF-2α-dependent expression of the intestinal epithelial divalent metal transporter 1 (DMT1 = NRAMP2) may be of interest, because they suggest that hypoxia or chemical HIF inducers could stimulate iron uptake by intestinal epithelial cells, which may perhaps lead to elevated serum iron and hepcidin levels [45].

### Post-Transcriptional Mechanisms May Contribute to Hepcidin mRNA Regulation

In HepG2 cells, DP exposure led to decreased hepcidin mRNA levels, but activated the hepcidin promoter, suggesting that the reduction of hepcidin mRNA expression is not due to transcriptional suppression, but at least partially caused by post-transcriptional mechanisms such as mRNA destabilization. Hepcidin promoter activation by DP in HepG2 cells could be completely prevented by HIF-1α knock-down, but was independent of the putative HREs in the hepcidin promoter (data not shown), suggesting that the marked HIF-1 activation by DP, which reproducibly exceeds that by hypoxia and DMOG, induces a factor which up-regulates hepcidin on the transcriptional level. At present we have no explanation for this observation. BMP-2,-4, and -6 expression patterns in HepG2 and Huh7 cells (Figure 8) could not explain the difference between the two hepatoma cell lines. Moreover, preliminary experiments showed that DP did not induce IL-6 in hepatoma cells, which was a further candidate mediator of hepcidin expression.

### Serum Components Are Required for Hepcidin Expression *In Vitro* and May Modulate Hepcidin Regulation

One of the most striking results of the present study was the rapid and marked decrease of hepcidin mRNA levels upon serum withdrawal. This effect was not rescued by supplementation of transferrin-bound iron, which indicates that other factors, e.g. BMPs, IL-6 and/or growth factors are necessary for the high basal hepcidin expression in HepG2 and Huh7 cells. A recent study demonstrated association of TfR2 with lipid rafts and mitogen-activated protein kinase signalling [36], which may hint at a convergence of growth factor, cytokine and TfR2 signal transduction pathways in hepcidin regulation and thus partially explain the effects of serum components on hepcidin expression. Presumably, not only the basal expression but also the hypoxic regulation of hepcidin may be modulated by a balance between hypoxia-regulated endogenous growth-factors and components of the fetal calf serum, since a change of the batch of serum used for cell culture also modulated the hypoxic hepcidin response and caused the high variability of the hypoxic hepcidin response as demonstrated in Figure 1. Identification of which components of fetal calf serum are necessary to maintain hepcidin expression, although beyond the scope of this study, may provide novel targets for future therapies able to modulate hepcidin levels in human disease. In the present study, we show by the use of protein kinase inhibitors that PI3 kinase activity is required for basal hepcidin expression in Huh7 cells, whereas inhibition of the MAP kinase pathway did not decrease, but rather increased hepcidin mRNA levels in Huh7 and HepG2 cells.

### The Regulation of TfR2 Expression Parallels Hepcidin Expression in Hepatoma Cells

A further novel finding of the present study was that, in contrast to BMPs, TfR2 expression was regulated in a pattern that matched hepcidin regulation: hypoxia slightly increased, whereas DP and DMOG decreased, TfR2 expression. TfR2 mutations have been reported to underlie hereditary hemochromatosis indicating a pivotal role for TfR2 in the transcriptional regulation of hepcidin (for review see [12]). Recently, Gao and co-workers demonstrated that in the presence of transferrin-bound iron (Fe2Tf), TfR1 is released from its interaction with HFE and replaced by the TfR2-Fe2Tf complex, though at an alternative binding domain of HFE [38]. Thus, HFE may transcriptionally activate hepcidin through TfR2 signaling, emphasizing the functional significance of TfR2 in hepcidin expression. The study also showed that HFE expression is low in HepG2 cells and overexpression is required to elicit clear Fe2Tf effects in HepG2 cells, which may explain the rather moderate effects of the experimental interventions observed in the present study.

Due to these limitations of cell culture models, we also cannot rule out that *in vivo* modulation of TfR1 expression by IRPs and/or HIF may regulate hepcidin expression.

### Determinants of Hepcidin Expression *In Vivo*: A Paradigm Shift?

Previous reports demonstrated that the decrease of hepcidin expression by experimentally induced anemia in animals is dependent on erythropoiesis, since it could be abolished or blunted by inhibitors of erythropoiesis such as irradiation, carboplatin, doxorubicin or EPO-blocking antibodies [46], [47]. These results suggested that hepcidin may respond to a signal arising from the erythropoietic activity itself and showed that anemia and tissue hypoxia *per se* were not sufficient to suppress hepcidin. Although liver-specific HIF-1α and VHL knock-out models rather suggested direct effects of HIF on hepcidin expression [21], the experiments presented did not exclude that the stimulation of erythropoiesis was the underlying cause of the observed effect on hepcidin. Growth differentiation factor 15 and BMP-6 have recently been implicated in hepcidin regulation in mice [14], [48], [49]. It is therefore conceivable that the balance and competition between factors of the transforming growth factor family, secreted by the expanding erythron and the liver may be critical in the transcriptional regulation of hepcidin.

However, a recent study in chronic kidney disease (CKD) patients, based on a novel immunoassay detecting the functional 25-amino acid hepcidin peptide, rather suggested that erythropoiesis may be the signal for hepcidin suppression [44]. More recently, erythropoietin administration to healthy humans (which would not be predicted to increase HIF activity) was observed to result in profound and prolonged suppression of circulating hepcidin which could not be attributed to changes in circulating iron or GDF-15 [50]. This indicates that, also in humans, neither hypoxia nor anemia are required for the suppression of hepcidin by erythropoiesis and challenges the idea that erythropoietin induction and hepcidin inhibition are *parallel* effects of HIF activation. While there is some evidence that erythropoietin may directly suppress hepcidin production in HepG2 cells in culture [51], the two collaborating groups of this manuscript could not reproduce these experiments (data not shown) and it seems more likely that a circulating factor released by the bone marrow is able to modulate hepcidin expression, not least because bone marrow ablation prevents suppression of hepcidin following erythropoietin administration in mice [47].

In keeping with the known limitations of cell culture models, our data show that hepcidin is not a direct negative target of HIF, but its expression can be modulated by a multitude of extrinsic factors and cell-autonomous regulatory pathways, some of which may be subject to regulation by HIF. While identification and pharmacological targeting of these factors may provide novel clinical therapies for the treatment of hemochromatosis or anemia of chronic disease (ACD), the pharmacological HIF inducers which are currently in clinical trials may exert suppressive effects on hepcidin as a consequence of the induction of erythropoiesis by increasing erythropoietin production and would therefore not be illogical treatments for ACD. Whether they are superior to EPO therapy in the treatment of ACD remains to be shown.
